# Haptic Meditation Enhancement Device (HMED): An Arduino-Based Multi-Sensor Real-Time Monitoring and Intervention Support System

**DOI:** 10.3390/s26134135

**Published:** 2026-07-01

**Authors:** Chuan-Wen Luo, Yang You, Xiao-Fan Huang, Hao Pan, Xin-Yang Zhang, Jia-Hui Wang, Ming-Run Wang, Abudusalamu Nuermaimaiti, Zhan-Yi You, Bo Zhang, Yan Zhang

**Affiliations:** 1 Spatial Human-Computer Interaction Laboratory, School of Architecture and Art, North China University of Technology, Jinyuanzhuang Road 5, Shijingshan District, Beijing 100144, China; youyang@mail.ncut.edu.cn (Y.Y.); huangxiaofan@mail.ncut.edu.cn (X.-F.H.); panhao@mail.ncut.edu.cn (H.P.); xinyangzhang@mail.ncut.edu.cn (X.-Y.Z.); wjh@mail.ncut.edu.cn (J.-H.W.); wangmingrun1@mail.ncut.edu.cn (M.-R.W.); abudusalamu@mail.ncut.edu.cn (A.N.); youzhanyi@mail.ncut.edu.cn (Z.-Y.Y.); 2 Beijing Historical Building Protection Engineering Technology Research Center, Beijing University of Technology, Beijing 100124, China; zy2021@emails.bjut.edu.cn

**Keywords:** haptic meditation enhancement device, sensor technology, mental health

## Abstract

As the pace of modern life continues to accelerate, the pressure participants face is growing, and mental health issues are becoming increasingly prominent. Against this backdrop, meditation, as a proven method for stress relief and relaxation, has garnered widespread attention. However, many people face challenges during meditation, such as difficulty entering a meditative state quickly or achieving sub-optimal outcomes. This is particularly true for beginners, who often struggle to accurately gauge the rhythm of meditation and thus fail to fully harness its regulatory effects on both body and mind. To address these issues, this study proposes a handheld meditation device. By making contact with the body via sensors, the device can measure multiple physiological metrics in real time, including skin conductance, electromyography, and heart rate. Based on these measurements, the device can monitor the user’s emotional fluctuations in real time. When emotional changes are detected, it uses the data to play music, release specific scents, or adjust lighting ambiance, thereby dynamically regulating the user’s psychological state. This helps users better immerse themselves in a meditative state and effectively enhances the benefits of meditation. This paper provides an in-depth analysis of the device’s design principles, detailing its hardware components—including various sensors and emotional regulation modules—and explaining the operational logic of its software algorithms. The effectiveness and reliability of the device were verified through rigorous experiments. The study also thoroughly examines the application prospects and potential value of this handheld meditation device, exploring new approaches and methods for the development of meditation technology and related equipment.

## 1. Introduction

### 1.1. Meditation and Mental Health

The fast pace and high stress of modern society have led to increasingly prominent mental health issues. As an ancient and effective method of psychological regulation, meditation can help participants reduce stress, improve concentration, and enhance emotional well-being [1]. Through self-regulation of the mind and body, and the conscious practice or training of physical relaxation or mental tranquility, meditation can have positive effects on both physiological and psychological aspects. Meditation offers a wide range of benefits: research indicates that long-term meditation can regulate the neuroendocrine system, lower blood pressure, alleviate physical pain, and boost immunity [2,3]. Psychologically, it can reduce negative emotions such as anxiety and depression, enhance psychological resilience, and help participants cope more calmly with life’s pressures. It can also improve focus and memory, making thinking clearer and more agile, and thereby boosting learning and work efficiency [4]. In daily life, setting aside 15 to 30 min each day for meditation practice can gradually bring about positive physical and mental changes, paving the way for a healthier, more balanced lifestyle. However, many beginners find it difficult to quickly enter a deep meditative state. Developing a haptic meditation enhancement device capable of real-time monitoring of physiological states and providing personalized adjustments holds significant practical value.

### 1.2. Literature Review

Meditation, as a time-honored mind–body practice, aims to guide individuals into a state of deep tranquility and focus through specific methods. It originated from the Vedic traditions of concentration (Dhāraṇā) and contemplation (Dhyāna), as well as early Taoist practices such as “sitting in forgetting” and “fasting of the mind.” Initially closely tied to religion and philosophy, it was used to explore the mind and pursue spiritual awakening [5]. Over time, meditation has gradually spread worldwide, becoming a practical skill used by the general public to improve physical and mental health.

In both clinical and non-clinical settings, meditation is recognized as a practice that enhances well-being and has demonstrated positive effects on conditions such as depression, addiction, and pain [6]. Meditation encompasses various conscious mental and cognitive activities, such as observation, focus, letting go, generation, imagination, and movement. These exercises can be performed in both formal and informal settings, based on the awareness of one’s own consciousness, and include mindfulness, mantra meditation, yoga, and tai chi, as well as deep meditative states such as jhana.

#### 1.2.1. Meditation Assistive Devices

Current research on meditation-support devices exhibits a highly interdisciplinary nature, spanning fields such as cognitive neuroscience, human–computer interaction, and biomedical engineering. It primarily focuses on technical directions including Electroencephalography (EEG) neurofeedback, VR-based immersive meditation, multi-modal perceptual interaction, intelligent signal classification, and non-invasive neurostimulation, featuring diverse approaches and a solid foundation in basic research.

Recent advances in artificial intelligence have also significantly promoted the development of emotion-recognition technologies. For example, STT-Net proposed a simplified temporal transformer architecture that effectively captures temporal dependencies in emotional signals while reducing computational complexity, demonstrating strong performance in emotion-recognition tasks [7]. In the field of visual affective computing, ARTriViT proposed a ViT-based Siamese neural network with triplet-loss optimization, providing an efficient framework for visual feature representation and classification in face-recognition applications [8]. Furthermore, Akçay and Oğuz reviewed recent progress in deep-learning-based speech emotion recognition, highlighting the effectiveness of convolutional neural networks, recurrent neural networks, transformers, and multimodal fusion strategies for emotion analysis [9]. These studies demonstrate the growing potential of intelligent emotion-recognition algorithms and provide important references for the development of adaptive affective computing systems. However, unlike these data-driven emotion-recognition approaches, the proposed HMED focuses on personalized physiological-state assessment and adaptive meditation intervention using lightweight physiological sensing and rule-based decision strategies, thereby emphasizing practicality, low deployment cost, and real-time usability in meditation-support scenarios.

EEG neurofeedback is the most intensively studied technical direction in the field of haptic meditation enhancement devices. It encompasses multiple levels, including the validation of fundamental neural mechanisms, clinical evaluation of wearable devices, and intelligent classification algorithms, and has formed a complete research chain spanning from basic mechanisms to clinical applications. Duda et al. provided scientific evidence confirming that alpha waves can serve as an objective neurofeedback indicator of meditation focus [10]. Kurek et al. overcame laboratory limitations and validated the feasibility of using lightweight mobile EEG devices to conduct neuroscience research on meditation in natural settings [11]. Lee et al. conducted the first randomized, double-blind, controlled clinical trial of the MAVE wearable neurofeedback device, demonstrating that neurofeedback-assisted meditation was significantly superior to sham-feedback meditation in reducing subjective stress [12]. Luctkar-Flude et al. directly compared a standard meditation app (Headspace) with the MUSE EEG feedback headband, demonstrating that MUSE significantly outperformed Headspace in improving perceived stress and state anxiety [13]. Usgaonkar et al. proposed a meditation EEG classification framework combining the Sea Lion Optimization (SLnO) algorithm with deep learning, enabling the automated differentiation of experts, novices, and control groups, and providing a practical technical solution for automated state assessment in smart-handed meditation-support devices [14].

Virtual reality (VR) technology offers a new avenue for experimentally manipulating self-perception in haptic meditation enhancement devices. Yang et al. validated the role of minimal body self-awareness in meditation through VR perspective manipulation and established the feasibility of using heart-evoked potentials (HEPs) as an objective indicator for assessing meditation depth [15]. Shin et al. found through comparative experiments that the design of natural scene content has a greater impact on meditation effectiveness than the VR hardware itself, providing a basis for the development of low-cost digital meditation products [16].

Multimodal haptic meditation enhancement devices have emerged as a new direction in the field of human–computer interaction in recent years. Cho et al. were the first to introduce contactless ultrasonic mid-air haptics into mindfulness meditation assistance, proposing four core design principles for haptic feedback [17]. Collaud et al. introduced the concept of “parametric meditation,” noting that appropriate coordination of multimodal stimuli can enhance the experience, but excessive layering can lead to sensory overload [18]. Bahrova et al. found that meditation elicits three primary physiological response patterns: arousal, balance, and relaxation. They established a ‘gold standard’ reference model for the ideal meditative state, which showed an average improvement of 19.3% (*p* < 0.05) after 30 training sessions, providing a quantifiable metric for the design of personalized biofeedback systems that can be embedded in wearable devices [19].

Additionally, Abellaneda-Pérez et al. systematically reviewed regulatory evidence integrating non-invasive brain stimulation (NIBS) with the neuroscience of meditation, proposing closed-loop adaptive stimulation guided by real-time EEG oscillations [20]. Abdoun et al. developed the LAMP scale, covering seven dimensions—context, volition, emotion, somatics, attention, cognition, and metacognition—advancing the evaluation of meditation effects from trait measurement toward dynamic process modeling [21]. Jylkkä et al. used a mixed-methods approach to compare the types of deep insights in psychedelic and meditation experiences, confirming that meditation can serve as a safe pathway to induce transformative experiences [22].

Compared to international research, Chinese studies on haptic meditation enhancement devices focus on clinical medical applications, emphasizing the validation of the therapeutic efficacy of mindfulness meditation interventions. While the technological platforms used are relatively traditional, these studies have accumulated high-quality, evidence-based data with local characteristics in specific clinical settings, and have made significant progress in neuroscientific methodologies and large-scale clinical trials.

Regarding the refinement of neurofeedback algorithms, Shang et al. employed convolutional neural networks (CNNs, including ShallowConvNet and DeepConvNet architectures) and support vector machines (SVMs) combined with Filter Bank Co-Spatial Pattern (FBCSP) features to identify the “state effects” (differences in EEG between meditation and resting states) and “trait effects” (changes in resting-state EEG before and after training) of short-term MBSR training in novice meditators. The study validated the advantages of CNN architectures in recognizing the meditative states of novice meditators, providing a crucial basis for the design of neurofeedback protocols for consumer-grade wearable EEG devices [23]. Regarding signal acquisition technologies for auxiliary devices, researchers are actively exploring the application potential of functional near-infrared spectroscopy (fNIRS) in meditation research. Qi et al. monitored dynamic changes in blood oxygen levels in the prefrontal cortex during standing meditation (Tai Chi Zhan Zhuang), a practice characteristic of traditional Chinese culture, revealing the relationship between prefrontal blood flow changes and the reorganization of brain functional networks [24]. A research team from Beijing Sport University combined mindfulness meditation intervention, fNIRS, and eye-tracking to confirm the beneficial effects of pre-competition mindfulness meditation on athletes’ attention control abilities [25]. Regarding large-scale clinical validation of neurofeedback-assisted meditation, a joint team from Peking Union Medical College and Peking University Sixth Hospital conducted the largest randomized controlled trial of neurofeedback-assisted mindfulness intervention in China to date. Jing et al. demonstrated that neurofeedback-assisted mindfulness-based intervention (NF-MBI) outperforms conventional mindfulness-based intervention (MBI) in both the short-term and long-term effects regarding the speed and magnitude of improvement in depression and anxiety [26]. In the field of cardiovascular disease management, Wu et al. designed a standardized mindfulness meditation intervention program for patients undergoing maintenance hemodialysis with in-dialysis hypertension (IDH). The study confirmed that the intervention significantly reduced blood pressure and improved quality of life, demonstrating its feasibility for implementation in primary care settings [27]. Deng and Xu demonstrated through an 8-week MBSR intervention that handheld meditation-support interventions can effectively alleviate academic stress and sleep disorders in adolescents [28].

In summary, while significant progress has been made in current research, numerous gaps remain in this field. For instance, the mechanisms underlying the synergistic effects of multimodal sensory stimulation remain unclear internationally; closed-loop regulation systems lack sufficient real-time responsiveness and precision; most studies focus solely on short-term intervention effects without long-term longitudinal tracking of neuroplasticity and behavioral changes; and existing models are predominantly based on Western populations, with a scarcity of research on cross-cultural adaptability. Research in China remains in its infancy, with relatively lagging technology; core hardware and algorithms rely on imports, and there are very few independently developed consumer-grade smart haptic meditation enhancement devices. Issues such as incomplete multimodal physiological sensing and closed-loop regulation mechanisms have not been effectively resolved.

#### 1.2.2. Physiological Parameter Monitoring Technology

Current physiological parameter monitoring technology is rapidly evolving toward multimodal integration, flexibility, intelligence, and scenario-specific applications. International research has established a complete research chain ranging from fundamental material mechanisms and interdisciplinary system integration to clinical translation. China, meanwhile, has achieved breakthroughs in novel flexible sensing materials, deep learning signal processing algorithms, and sports and health applications. However, both sides still face common challenges in clinical standardization and validation, multimodal signal decoupling, and the practical implementation of research outcomes.

Wearable sensors form the core hardware foundation of physiological parameter monitoring systems. In recent years, significant international progress has been made in flexible sensing materials and multimodal integration. Homayounfar et al. systematically reviewed five sensor mechanisms—piezoresistive, piezoelectric, capacitive, triboelectric, and transistor-based—and defined key performance metrics such as sensitivity, stretchability, and response time, providing a standardized framework for the selection and design of wearable sensors [29]. Regarding multimodal integrated systems, Assaad et al. developed an Internet of Things (IoT)-enabled wearable multimodal biosensing device that integrates 14 sensors to simultaneously collect 18 physiological, emotional, and cognitive metrics, including ECG, EEG, electromyography (EMG), PPG, SpO_2_, blood pressure, and blood glucose (exhaled acetone method), with most metrics achieving an accuracy rate exceeding 90% [30]. With the rapid development of deep learning technologies, intelligent algorithm-based physiological signal processing has become a research hotspot in the field of physiological parameter monitoring. Mahmud et al. proposed NABNet (Nested Attention-Guided Bidirectional Convolutional LSTM Network), achieving a mean absolute error (MAE) of 2.63 mmHg for systolic blood pressure (SBP) and 1.09 mmHg for diastolic blood pressure (DBP) in non-invasive continuous blood pressure monitoring on the MIMIC-III dataset, meeting AAMI standards and BHS Class A accuracy requirements [31]. Umer et al. introduced physiological monitoring to construction sites, achieving a 98.5% accuracy rate in worker fatigue classification using a Bi-LSTM model [32]. In the clinical application domain, Pundi et al. integrated the FDA’s 2023 guidelines on “Digital Health Technologies for Remote Data Collection in Clinical Studies” with cardiovascular safety assessment requirements, emphasizing the “human–machine collaboration” principle and the necessity of dynamic physiological baselines. They provided a regulatory science framework for remote cardiac safety monitoring, representing one of the most authoritative expert consensuses in this field internationally [33].

Chinese research, meanwhile, demonstrates significant strengths in material innovation, algorithmic breakthroughs, and specialized applications. Regarding novel flexible sensing materials, Wang et al. developed a highly sensitive flexible pressure sensor based on cellulose acetate carbonate (CCA) and thermoplastic polyurethane (TPU) nanofiber membranes, achieving a sensitivity of up to 101.22 kPa^−1^, a lower detection limit of 5 Pa, and a response time of just 20 ms. The sensor demonstrated excellent durability after 10,000 cycles and can effectively monitor various physiological signals, including pulse waves, respiratory rate, and joint movement [34]. Hsu et al. developed an ultra-soft piezoelectric muscle patch sensor (MPS) that enables non-invasive monitoring of three types of muscle contractions—isometric, concentric, and eccentric—by measuring changes in the peripheral length of the physiological cross-sectional area (PCSA). It can also identify muscle fatigue through tremor signals at 7–15 Hz, with a fatigue detection accuracy ranging from 81.4% to 95.7% [35]. In terms of signal denoising and enhancement, Liu et al. proposed the PMD-Net multi-cascade denoising network, which improved the signal-to-noise ratio (SNR) of physiological micro-vibration signals to 25.38 dB under extremely low SNR conditions (−14.48 dB), providing technical support for the initial screening of obstructive sleep apnea in home settings [36]. Wang et al. developed a dual-task learning framework (constrained regression) based on 24 GHz radar, utilizing a multi-scale ResNet to extract spatiotemporal features and a three-layer bidirectional gated recurrent unit (GRU) to model the temporal continuity of blood pressure changes, thereby achieving non-contact continuous blood pressure monitoring that meets the BHS Class A standard [37]. The application of wearable physiological monitoring in the fields of sports science and mental health assessment is receiving increasing attention. Chen et al. comprehensively reviewed the roles of wearable physiological monitoring devices, summarizing the applications of physiological signals such as ECG/heart rate variability (HRV), EEG, fNIRS, EMG, and PPG, as well as their multimodal fusion technologies, in monitoring psychological states such as depression, anxiety, and stress [38]. Wang demonstrated the practical value of multimodal wearable devices in optimizing exercise training loads, promoting recovery, and managing psychological stress [39].

In summary, internationally, a complete research chain has been established in the field of physiological parameter monitoring technology, spanning from fundamental material mechanisms and interdisciplinary system integration to clinical translation. In China, substantial empirical evidence has been accumulated in areas such as novel flexible sensing materials, deep learning-based signal enhancement algorithms, and non-contact monitoring technologies. Novel materials, deep learning, non-contact radar technology, and multimodal fusion have become the core technological pathways in this field. However, existing research generally lacks large-scale clinical validation, suffers from low device standardization, and requires improvements in signal robustness under real-world dynamic scenarios; these are key areas for future research breakthroughs.

#### 1.2.3. Limitations of Existing Research and Innovations of This Study

Although significant progress has been made in haptic meditation enhancement devices and physiological parameter monitoring technologies—giving rise to diverse technical approaches such as EEG neurofeedback, VR immersive experiences, and multimodal sensory interaction—existing research still has numerous shortcomings that severely hinder the widespread adoption and clinical translation of these technologies. Research in physiological sensing systems faces a dichotomy between “high cost and low adoption” and “low cost and low accuracy.” While mainstream EEG neurofeedback devices offer high accuracy, they are cumbersome to wear and prohibitively expensive, making them unsuitable for the general public’s daily needs. Conversely, low-cost single-sensor devices suffer from insufficient signal dimensions and poor resistance to interference, and consumer-grade multimodal fusion algorithms lack optimization specifically tailored to meditation scenarios. Second, multimodal feedback mechanisms lack systematic design. Most devices employ a simple sensory stimulus overlay model, which can easily lead to sensory overload. Furthermore, there is no consensus on the optimal ratio of different modalities, their timing, or their synergistic mechanisms, resulting in insufficient real-time responsiveness and accuracy in closed-loop feedback. Additionally, there is a severe mismatch between device form factors and usage scenarios. Head-mounted devices are cumbersome to wear and carry a risk of motion sickness, while desktop devices lack portability. Complex app interaction processes conflict with the low-distraction experience required for meditation, and there is a particular lack of user-friendly design for beginners. Furthermore, existing models are mostly based on Western populations and lack localization; most studies focus only on short-term intervention effects and rely excessively on subjective scale assessments, and there is a severe shortage of consumer-grade products independently developed in China.

Compared with representative consumer-grade meditation and biofeedback systems, the proposed HMED exhibits several distinctive characteristics. Commercial meditation applications such as Headspace primarily rely on guided audio content and self-directed user participation, lacking real-time physiological sensing and adaptive intervention mechanisms. Wearable physiological monitoring devices and biofeedback platforms, including products such as Muse and other EEG-based neurofeedback systems, provide physiological-state monitoring and feedback functions but often depend on specialized sensing hardware, higher implementation costs, or limited sensory feedback modalities. In contrast, the proposed HMED integrates skin conductance response (SCR), EMG, and heart-rate (HR) sensing with adaptive multimodal feedback involving light, sound, and scent within a compact handheld device. Furthermore, the system adopts a personalized baseline-referenced assessment strategy and closed-loop intervention framework, enabling real-time physiological monitoring and adaptive regulation while maintaining low deployment cost, ease of use, and suitability for everyday meditation practice. These characteristics distinguish the proposed system from existing consumer-grade meditation aids and physiological biofeedback devices.

To address these research gaps, this study designed and developed a handheld meditation-support device based on multimodal physiological sensing, innovatively and systematically resolving the key shortcomings of existing devices. This study abandons traditional high-cost EEG-based approaches and instead develops a low-cost physiological monitoring system based on multidimensional physiological signals, including SCR, EMG, and HR. The proposed system supports real-time assessment of relaxation-related physiological changes and provides personalized meditation interventions through adaptive multimodal feedback. A dynamic hierarchical classification model based on Arduino-sensor-derived personal resting baselines was established. By evaluating emotional states based on relative changes rather than fixed thresholds, the model effectively eliminates recognition errors caused by individual physiological variations. The study proposes a three-tiered progressive multimodal collaborative feedback mechanism involving light, sound, and scent. This mechanism automatically adjusts the intensity and mode of intervention according to the user’s real-time physiological state, achieving adaptive closed-loop regulation while preventing sensory overload. The device features a handheld, all-in-one design that requires no complex electrodes or smartphone apps; it is ready for use immediately upon activation, significantly lowering the barrier to entry and expanding its range of applications. The study established a validation system combining objective and subjective measures, simultaneously collecting three physiological indicators alongside subjective data from the Self-Rating Anxiety Scale (SAS) and the Perceived Stress Scale (PSS). Through pre-post controlled experiments, the intervention effects of the device were comprehensively evaluated. This research not only provides meditation beginners with a low-threshold, highly user-friendly support tool but also offers a technical paradigm for the development and evaluation of consumer-grade smart health devices.

### 1.3. Design Philosophy of the Device

#### 1.3.1. Physiological Parameters and Psychological State

Changes in human psychological states do not occur in isolation at the brain level but are accompanied by synchronous changes in physiological signals across multiple systems throughout the body. The autonomic nervous system (ANS), serving as a bridge between the central nervous system and peripheral organs, plays a central role in emotional regulation and stress response. When an individual experiences different psychological states—such as stress, anxiety, or relaxation—the sympathetic and parasympathetic branches of the ANS exhibit distinct activation patterns, leading to corresponding changes in various peripheral physiological indicators, including HR, skin conductance, and muscle tension [40]. These changes in physiological signals are objective, real-time, and difficult to control subjectively, making them ideal physiological markers for assessing psychological states. Based on this theoretical foundation, this study selected three physiological indicators—galvanic skin response (GSR), EMG, and HRV—to construct a multimodal physiological sensing system for the real-time monitoring and dynamic assessment of users’ psychological states.

GSR, also known as SCR, is the core fast-changing component of Electrodermal Activity (EDA) and reflects the instantaneous excitation state of the sympathetic nervous system. At rest, human sweat gland activity is calm, and GSR values remain within an amplitude threshold of 0.2–0.5 μS, corresponding to a state of psychological calm and relaxation. When an individual faces stress or experiences anxiety, the sympathetic nervous system is activated, sweat gland secretion increases, and the water content in the skin’s stratum corneum rises, leading to a decrease in skin resistance and an increase in electrical conductivity. If the skin conductance value remains ≥0.8 μS for 10 s, this can be preliminarily interpreted as a significant emotional fluctuation. This change is not under the control of conscious awareness and can objectively reflect the degree of psychological tension. Research by Bahrova et al. further confirms that skin conductance is one of the key indicators for distinguishing among the three physiological response patterns of meditation arousal, equilibrium, and relaxation, with its trends showing a high degree of consistency with subjective experiences of relaxation [19]. By detecting real-time changes in palm skin conductance, this study uses it as the primary signal for assessing the user’s emotional arousal level, making it one of the core parameters for emotional monitoring in this device.

Complementing the emotional arousal monitoring function of skin conductance, EMG records the electrical activity generated during muscle fiber contraction, indirectly reflecting the state of physical tension induced by psychological stress. Psychological stress and anxiety not only trigger emotional responses but also lead to sustained muscle tension. During psychological relaxation, skeletal muscles are in a state of low tension, and EMG signal amplitudes are low, typically <2 μV, whereas negative emotions such as anxiety and irritability lead to unconscious muscle contractions—such as tightness in the shoulder and neck muscles or facial muscle tension—causing EMG signal amplitudes to rise significantly, reaching over 5 μV. In long-term meditation practitioners, EMG signals exhibit a sustained, low-amplitude, and stable pattern during states of deep concentration, which correlates positively with psychological relaxation and focus. In meditation settings, EMG metrics can compensate for the limitations of EEG and HR, which reflect only autonomic nervous system activity, by comprehensively assessing the user’s state of relaxation at the somatic level. This is particularly suitable for identifying unconscious muscle tension—a common issue among beginners—and provides real-time somatic triggers for closed-loop interventions.

HRV is a key indicator for assessing autonomic balance and psychological state. HRV reflects the heart’s ability to self-regulate, with its core physiological mechanism lying in the dynamic balance regulation of the sympathetic and parasympathetic nervous systems by the Central Autonomic Network (CAN). At rest, the HR of a healthy adult typically remains between 60–80 bpm, a state dominated by the vagus nerve that corresponds to psychological relaxation and emotional stability. When an individual experiences emotions such as anxiety or tension, sympathetic nervous system activation causes the HR to accelerate, sustaining a rate of ≥90 bpm for 10 s, while HRV decreases—that is, the variability in heartbeat intervals diminishes. This change directly reflects a decline in psychological regulatory capacity. Conversely, during deep meditation, the HR tends to slow, and HRV increases accordingly, reflecting enhanced autonomic nervous system regulation and a more stable psychological state. In meditation intervention studies, regular meditation practice has been shown to improve HRV parameters and reduce HR and stress indices. In this study, HR metrics are primarily used to assess the user’s overall arousal level and relaxation effects, and are used in conjunction with skin conductance and electromyography to evaluate the user’s current autonomic nervous system state and depth of meditation.

Although individual physiological indicators can reflect changes in psychological state within specific dimensions, each has certain limitations. By simultaneously collecting these three physiological indicators, the study leverages the synergistic advantages of multimodal fusion to characterize the user’s psychological state, emotional arousal level, stress status, and degree of relaxation from multiple dimensions. These metrics corroborate and complement one another, collectively forming a more comprehensive and stable physiological assessment system. This ensures that the device can objectively and in real-time capture emotional fluctuations during the user’s meditation process, providing reliable input for subsequent dynamic feedback regulation.

#### 1.3.2. The Regulatory Role of External Interventions on Psychological States

The regulatory effect of external sensory stimuli on psychological states has been confirmed by a large body of research. Single-channel sensory stimuli—such as visual, auditory, olfactory, and gustatory inputs—can significantly influence mood and depressive states. This influence is bidirectional: sensory input can modulate mood, while emotional states, in turn, alter sensory perception [41]. Based on this theoretical framework, the device employs multimodal intervention through three channels—music, scent, and lighting—whose respective neurophysiological mechanisms differ yet may produce synergistic effects.

Music influences the limbic system and the autonomic nervous system through elements such as rhythm, melody, and harmony, thereby achieving emotional regulation. There is a clear coupling relationship between the tempo of music and the body’s physiological rhythms. Slow tempos (40–60 bpm) are close to resting HR, guiding respiration and HR to synchronize with this frequency and reducing sympathetic nervous system activity. In a randomized controlled trial involving patients with dental anxiety, both classical music and Turkish folk music significantly reduced systolic and diastolic blood pressure as well as HR, indicating that music has a direct alleviating effect on physiological stress [42]. Conversely, excessively fast musical rhythms increase blood pressure variability, which is detrimental to maintaining a state of relaxation [43]. Although natural sounds (such as rain, flowing streams, and birdsong) lack melody and harmony in the traditional sense, their random yet steady acoustic characteristics produce a unique relaxing effect, significantly lowering HR, blood pressure, and respiratory rate, while also demonstrating advantages in alleviating subjective stress [44]. Therefore, the device employs slow-tempo relaxation music and natural soundscapes as its core auditory interventions.

Odor molecules project directly from the olfactory epithelium to limbic system structures such as the amygdala and hippocampus, bypassing the thalamus as an intermediary. Consequently, their emotional regulatory effects take effect rapidly and exert a direct influence. Common scents that aid relaxation and meditation primarily fall into three categories: forest scents (fir, pine), herbal and floral scents (lavender, chamomile), and earthy scents. Inhalation of linalool from lavender or 2-phenylethanol from roses significantly reduced anxiety-like behaviors in mice during elevated plus maze and open field tests [45]. This indicates that plant scents do not merely work through psychological suggestion but have a clear neurochemical basis. The device utilizes natural extracts or safe analogues of the aforementioned scents, controlled by a micro-atomization module to regulate release, thereby preventing olfactory fatigue and achieving gentle, sustained emotional regulation.

Light influences the suprachiasmatic nucleus (SCN) and the pineal gland via the retina–hypothalamus pathway, thereby regulating melatonin secretion and autonomic nervous system balance, and exerting a modulatory effect on mood and depression. Light with a low color temperature (2700–3000 K, warm yellow/amber) can reduce an individual’s tendency toward negative emotional responses and help alleviate feelings of tension and anxiety [46]. The color of interior walls also affects melanopic lux levels through light reflectance, which may suppress melatonin secretion and disrupt sleep rhythms; in contrast, warm-toned walls can effectively reduce this risk [47]. Regarding brightness, excessively high levels (>1000 lx) increase alertness and physiological arousal, which are not conducive to relaxation; conversely, excessively low levels (<30 lx) may induce drowsiness or restlessness. The optimal illuminance for meditation is generally 30–150 lx; an initial level of 50–100 lx can be used to aid adaptation, and during deep meditation, this can be reduced to 30–50 lx to minimize visual interference [48]. Regarding flicker frequency, low-frequency flicker (0.5–2 Hz) can guide breathing rhythms to synchronize with this frequency, thereby reducing anxiety. Therefore, the device primarily uses a constant-light mode, employing low-amplitude, low-frequency soft flicker in different hues only when assistance with breathing guidance is needed to aid regulation.

In summary, the three intervention methods—music, scent, and lighting—act on the emotional regulation system through the auditory, olfactory, and visual pathways, respectively. Their mechanisms of action have distinct focuses but are complementary. This device will dynamically combine these three types of stimuli based on real-time physiological feedback to achieve personalized, multimodal handheld meditation-support.

## 2. System Requirements and Design

The core design objective of this device is to enable real-time monitoring of the user’s physiological state during meditation and to dynamically adjust multimodal sensory outputs—such as music, scent, and lighting—based on the monitoring results, thereby assisting the user in rapidly entering and maintaining a deep meditative state. To achieve this goal, the system’s main controller must be capable of reliably driving multiple analog and digital sensors—including those for GSR, EMG, and HR—processing the collected data in real time to classify emotional states, and synchronously controlling the output of multiple actuators, such as the audio playback module, the atomization control module, and the PWM dimming module. Therefore, the selection of the main control platform must strike an optimal balance between development friendliness, interface resources, processing performance, and ecosystem maturity.

### 2.1. Basis of the System Design

Among current mainstream embedded development platforms, the 51 series microcontrollers feature a simple architecture but have limited performance and scarce analog interface resources, making it difficult to meet the requirements for simultaneous multi-channel sensor data acquisition and real-time algorithm execution. The STM32 series offers excellent performance and abundant peripheral resources, making it the mainstream choice for industrial-grade products; however, its development environment configuration is cumbersome, the learning curve is steep, and the development cost is relatively high for prototype research aimed at functional verification. Although the ESP series offers significant advantages in wireless connectivity, its analog signal processing capabilities are relatively limited, and it suffers from insufficient ADC precision in scenarios involving multi-channel analog sensor acquisition.

In contrast, the Arduino platform, with its open-source hardware design, standardized interface specifications, and highly active developer ecosystem, has become the ideal choice for prototyping and multi-sensor integration applications (Table 1). The Arduino IDE provides full support for C/C++ and includes a large number of well-tested open-source libraries, allowing direct access to drivers and signal processing algorithms for GSR, pulse oximetry, and EMG sensors, which significantly reduces development time. Its cross-platform development environment supports Windows, macOS, and Linux systems and forms a complete toolchain ecosystem with design and data visualization tools such as Fritzing and Processing.

After selecting the Arduino platform, further screening of specific models is conducted. The Arduino UNO R3 (Arduino Srl, Ivrea, Italy) is based on the ATmega328P microcontroller (Microchip Technology, Chandler, AZ, USA), operates at a clock frequency of 16 MHz, and features 6 10-bit precision ADC analog input channels, 14 digital I/O pins (6 of which support PWM output), one hardware I^2^C interface, and one hardware SPI interface, as well as 16 KB of Flash and 2 KB of SRAM. These interface specifications fully meet the signal acquisition and actuator control requirements of this device. The analog voltage signal from the GSR sensor can be directly connected to the ADC channel; the HR sensor (MAX30102, Analog Devices (Maxim Integrated), Wilmington, MA, USA) communicates with the main controller via the I^2^C bus; the amplified EMG analog signal is connected to a dedicated ADC channel; and the MP3 audio, odor diffusion module, and LED dimming module occupy digital I/O and PWM output pins, respectively. The hardware specifications of the Arduino UNO R3 are shown in the table (Table 2).

In summary, the Arduino UNO R3 development board was selected as the core controller for this device. Although its clock frequency and memory capacity are lower than those of the STM32 (STMicroelectronics, Geneva, Switzerland) series, its processing power fully meets the system requirements for this device. The core algorithms of this device primarily involve threshold comparison and sliding window filtering, with computational complexity at a low to medium level. Regarding sensor sampling rates, the target sampling frequencies for both GSR and HR signals are within 100 Hz, far below the maximum sampling rate supported by the 16 MHz clock frequency. Therefore, the Arduino UNO R3 does not present any performance bottlenecks in this application scenario. Its mature open-source ecosystem and extremely low development barrier, on the contrary, constitute significant engineering advantages, effectively shortening the prototype validation cycle and enhancing the system’s reproducibility and scalability.

The response times of each sensor and actuator module are shown in the table (Table 3). The physiological signal acquisition and processing loop maintains a real-time response latency of less than 200 ms, which is crucial for accurately capturing rapid emotional fluctuations. However, the actuation latency varies significantly depending on the specific output module (as detailed in Table 3). While electronic actuators (light, sound) respond rapidly, the scent diffusion module exhibits a longer response time due to the physical atomization process.

During meditation training and mind–body relaxation, physiological signals such as EMG, GSR, and HR exhibit significant and rapid changes, serving as core indicators for assessing emotional fluctuations, stress levels, and overall physical and mental states. Therefore, the core design objective of this device is to achieve synchronous, real-time, and stable acquisition of three key physiological signals—EMG, GSR, and HR—and to construct a guided, adjustable, and interactive system for meditation, relaxation, and coordinated physiological regulation through multimodal feedback mechanisms such as ambient lighting, soothing audio, and scent diffusion.

**Figure 1:**
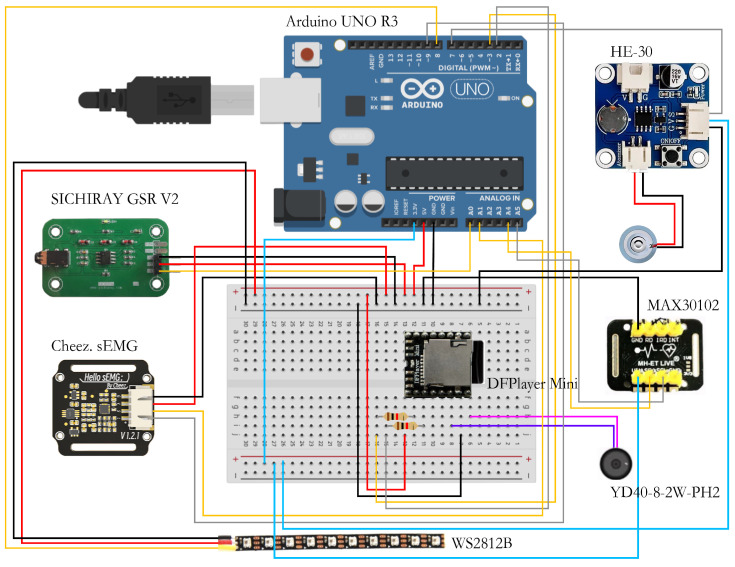
Electronic circuit of the meditation aid. The device integrates a GSR sensor (GSR V2), an EMG sensor (Cheez. sEMG), and an HR sensor (MAX30102); the letters next to the components in the figure indicate their specific models. The VCC pins of each sensor are connected to the development board’s 5 V or 3.3 V output, and the GND pins are connected to the board’s GND. The HR sensor is connected via an I^2^C digital bus, while the analog outputs of the GSR and sEMG sensors are connected to ADC channels. The three types of output modules are assigned to digital I/O or PWM pins. In the diagram, red wires represent the 5 V power supply, blue wires represent the 3.3 V power supply, and black wires represent ground; wires of other colors are used to distinguish different signal lines.

This haptic meditation enhancement device integrates high-sensitivity EMG, GSR, and HR sensors to continuously and non-invasively capture the user’s physiological relaxation signals. The collected data is presented intuitively through a multimodal feedback unit: ambient lighting, soothing audio, and scent diffusion correspond to different relaxation states and dynamically adjust their intensity based on real-time physiological analysis, providing users with clear, immediate, and immersive feedback on their physical and mental relaxation status (Figure 1).

### 2.2. Choice of Hardware

The manufacturing budget for this device consists of multiple components, with the total budget for the basic module components amounting to approximately $44.83 (Table 4). The cost of expansion components fluctuates with market prices, but the total cost will not exceed $80.

### 2.3. Open-Source Programming

**Figure 2:**
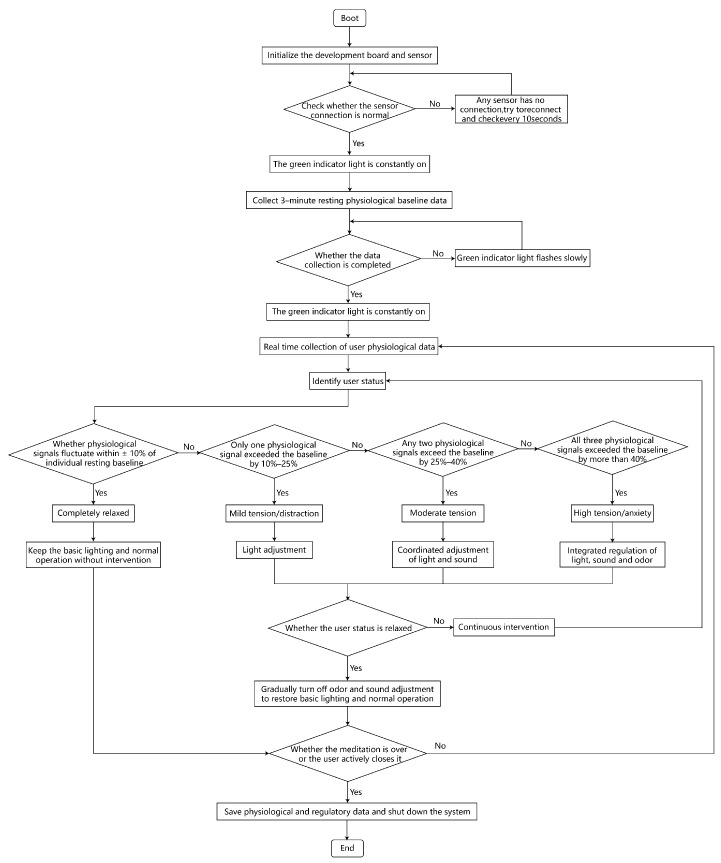
Flowchart of the haptic meditation enhancement device.

The embedded program for this device was developed using the Arduino IDE 1.8.19, an official cross-platform integrated development environment (IDE) provided by Arduino. It is open-source and available free of charge under the GNU General Public License (GPL), and supports Windows, macOS, and Linux operating systems. The device was primarily developed using the C/C++ programming languages, and efficient embedded development was achieved through the Arduino core library and low-level register operations. The overall program flow is described in detail below (Figure 2).

It should be noted that the proposed system does not employ a machine-learning classification framework. Instead, emotional-state assessment is performed using a personalized baseline-referenced rule-based decision strategy derived from variations in GSR, EMG, and HR signals. The system evaluates physiological changes relative to each user’s baseline condition and subsequently triggers corresponding multimodal feedback interventions.

## 3. Functional Validation

The haptic meditation enhancement device designed in this study aims to provide real-time physiological monitoring and multimodal feedback for both beginners and experienced practitioners, helping users better enter a relaxed state and thereby enhance the effectiveness of meditation.

### 3.1. Experimental Design

To ensure the scientific validity of the device in terms of sensor data accuracy, the effectiveness of feedback regulation, and overall system reliability, we designed a series of experiments.

A total of 30 healthy volunteers (aged 22–30 years) participated in the intervention evaluation study. Participants were randomly assigned to either the intervention group (*n* = 15) or the control group (*n* = 15). Individuals with diagnosed psychiatric disorders, sleep disorders, current use of medications affecting physiological responses, or other conditions that could interfere with meditation performance or physiological measurements were excluded from participation. Information regarding prior meditation experience was collected before the experiment, and no participant reported formal long-term meditation training. It should be noted that the sensor-validation experiments and the intervention-effectiveness evaluation were conducted using participants drawn from the same study cohort. Specifically, the five volunteers involved in the GSR validation experiment were part of the same cohort of 30 participants who completed the intervention evaluation, physiological measurements, SAS assessment, and PSS assessment.

We validated the data acquisition validity, functionality, and responsiveness of the device’s sensors. To this end, we employed a combined approach of self-validation and task-related validation, using an Arduino UNO R3 microcontroller to collect physiological monitoring data to verify the signal reliability and physiological validity of the GSR, EMG, and HR sensors. By collecting physiological parameters and assessing response consistency during resting, emotionally induced, and meditative states, we evaluated the measurement validity and dynamic response capabilities of the device’s sensors in real-world usage scenarios. We recorded GSR signals using the SICHIRAY GSR V2 sensor through baseline stability and evoked response tests. By inducing emotional arousal with a stress-inducing video, we verified the consistency between the increase in the amplitude of the SCR and subjective emotional ratings. We verified the ability of the (Cheez sEMG sensor) to clearly capture surface EMG signals through noise assessment and active contraction tests. We verified the HR sensor’s (MAX30102) reliable capture of HR signals through resting-state signal stability assessments and HR variability changes induced by a physiological task (deep breathing). All sensors demonstrated good signal-to-noise ratios and repeatability.

It is important to clarify the distinct objectives of our validation phases. The sensor-specific validation experiments (Section 3.2, Section 3.3 and Section 3.4) were conducted using music-only intervention. This was a deliberate design choice to isolate the physiological response to auditory stimuli and verify the baseline sensitivity of the GSR, EMG, and HR sensors without the confounding variables of light or scent. In contrast, the full-device intervention phase aims to evaluate the synergistic effects of the multimodal environment (music, light, and scent combined). Therefore, while the sensor validation establishes the reliability of data acquisition under controlled auditory stimulation, the subsequent intervention results reflect the holistic impact of the device’s integrated feedback system.

The regulatory effects of the multimodal feedback mechanism on the user’s physiological state. We established an experimental group (using this device for meditation) and a control group (using traditional meditation methods without any feedback intervention), and simultaneously collected physiological monitoring data from both groups of participants. By analyzing the amplitude of fluctuations, recovery speed, and changes in HRV of physiological indicators during meditation in both groups, we evaluated the effectiveness of the device’s three-tiered progressive feedback (light, sound, and scent) in improving the users’ state of relaxation.

Ultimately, we tested and validated the accuracy of the device’s dynamic regulation based on physiological feedback. In an experimental environment simulating real-world meditation scenarios, we established conditions with varying degrees of emotional fluctuation (e.g., mild tension, moderate tension, high anxiety) by playing tense video clips or assigning timed mental arithmetic tasks to induce emotional changes in participants. We recorded physiological fluctuations detected by sensors alongside the device’s triggered feedback responses (music playback, lighting adjustments, and scent diffusion). The subjective reports were used as a supplementary reference to evaluate whether the physiological trends detected by the device and the corresponding multimodal feedback responses were generally aligned with participants’ perceived relaxation states during meditation.

We comprehensively accounted for all factors that could potentially influence the experimental results, including ambient temperature, humidity, noise, and the participants’ age, gender, and meditation experience levels. Through random grouping, blinded outcome assessment, and rigorous baseline consistency checks, we minimized experimental error. Due to the perceptible nature of the multimodal interventions (music, light, and scent), participant blinding was not feasible. However, the outcome evaluation and data analysis procedures were conducted without reference to group allocation information to minimize potential assessment bias.

### 3.2. Validation of the Skin Conductance Sensor

The core of this research system is the monitoring of the user’s physiological parameters to assess emotional arousal, tension, and relaxation levels. These measurements serve as the primary basis for determining the depth of meditation and triggering adjustments to music, scent, and lighting. The GSR sensor is directly regulated by the sympathetic nervous system, reflecting sweat gland activity and emotional fluctuations without being influenced by conscious control. It is one of the gold standard indicators for assessing meditation states. Therefore, conducting precise and stable functional validation of the GSR sensor is crucial for ensuring the device’s emotional recognition and adaptive regulation capabilities. The study selected the SICHIRAY GSR V2 sensor module, which integrates high-precision signal amplification circuits and noise suppression units. Based on the principle of skin conductance changes, it non-invasively and in real-time measures skin conductance (μS). Its compact size, low power consumption, and strong resistance to interference make it highly suitable for embedding in haptic meditation enhancement devices for long-term, stable data collection. The sensor parameters are as follows (Table 5).

To guarantee the physiological validity of this experiment, a calibration process was first performed to convert the raw ADC values into standard conductance units (μS). Based on the SICHIRAY GSR V2 specifications (Table 5), the conversion formula is defined as:

(1) $$G=\frac{{ADC}_{raw}}{1024}\times \frac{V_{ref}}{R_{series}}$$

where V*_ref_* is the reference voltage (5 V) and R*_series_* is the internal series resistance of the sensor module.

**Figure 3:**
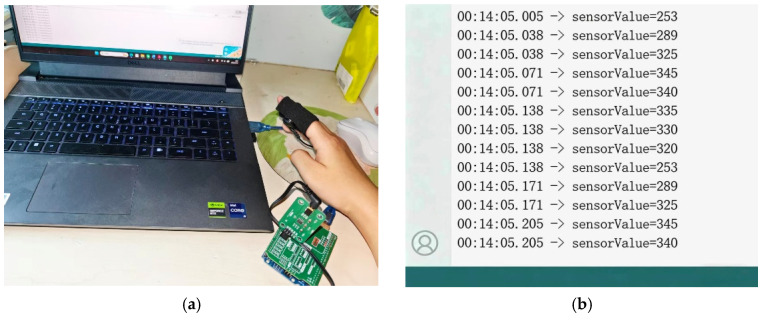
Validation of the GSR sensors. (**a**) Sensor functionality test; (**b**) data recording from the serial monitor.

To verify the accuracy, stability, and sensitivity to states of relaxation of the SICHIRAY GSR V2 sensor when collecting data in meditation scenarios, this study designed a stepwise validation experiment. The experiment was conducted in a standardized indoor laboratory (temperature 22–25 °C, relative humidity 40–60%) with five healthy volunteers (aged 22–30 years, with no skin diseases). The experiment was divided into three phases: resting baseline acquisition, unguided meditation, and guided meditation. During all phases, sensors were worn on the fingertips of the index and middle fingers of the same hand to ensure consistent contact pressure and position (Figure 3).

**Figure 4:**
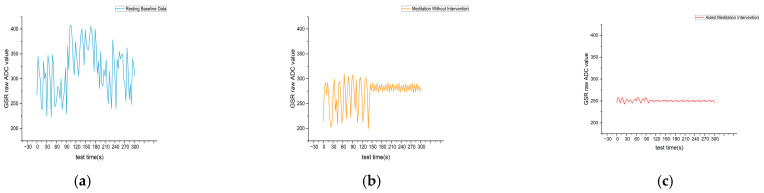
SICHIRAY GSR V2 accuracy validation data. (**a**) Initial physiological skin potential data of subjects wearing the SICHIRAY GSR V2 device at rest. (**b**) Physiological skin potential data of subjects wearing the SICHIRAY GSR V2 device during seated meditation. (**c**) Physiological skin potential data of subjects wearing the SICHIRAY GSR V2 device during seated meditation with intervention.

**Figure 5:**
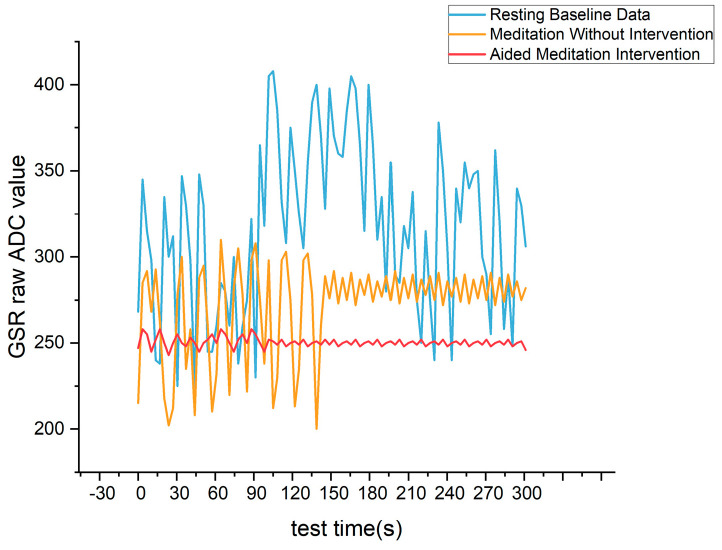
Data after SICHIRAY GSR V2 fitting. Fitted skin potential data of subjects wearing the SICHIRAY GSR V2 under resting state in non-intervened meditation and intervened meditation conditions.

During the experiment, subjects sat quietly for 10 min to stabilize their physiological and emotional states. Subsequently, resting skin conductance data were continuously collected for 5 min (sampling rate: 1 Hz), and the mean ADC, amplitude of fluctuations, and numerical range were recorded to serve as the physiological baseline for subsequent comparisons (Figure 4). Under the same environmental conditions, subjects performed 5 min of standardized meditation. No music or feedback interventions were provided during the experiment, and skin conductance data were collected simultaneously to serve as the non-intervention meditation data. During the intervention meditation, soothing background music (e.g., piano music, volume approximately 40 dB) was played continuously, and GSR data was collected synchronously for 5 min. Regarding the electrodermal activity, as shown in Figure 5, the GSR values of the experimental group exhibited a significant downward trend during the meditation session (from M = 2.43 μS, SD = 100 at baseline to M = 1.75 μS, SD = 0.10 post-intervention). A paired *t*-test confirmed that this reduction was statistically significant (t(29) = 6.45, *p* < 0.001), with a large effect size (Cohen’s d = 1.18) and a relative decrease of approximately 26.5% from baseline, indicating reduced sympathetic nervous system activity and a transition to a deep relaxation state.

Similarly, physiological consistency was observed in heart rate data (from M = 78.4 bpm, SD = 6.2 at baseline to M = 65.1 bpm, SD = 5.8 at post-intervention; t(29) = 8.73, *p* < 0.001, Cohen’s d = 1.59). These physiological changes were consistent with the subjective reports from the psychological scales (*p* < 0.01).

Based on the calibrated data presented in Table 6, a clear graded pattern is observed. The system transitions from a high mean conductance of approx. 2.43 μS (corresponding to ~340 ADC) in the resting state, decreases to approx. 1.93 μS (~270 ADC) during meditation without intervention, and reaches a low mean value of approx. 1.75 μS (~250 ADC) in the music-intervention meditation group. Compared to the resting baseline, unassisted meditation reduced the mean EDA by approximately 20.6%, while music-assisted meditation further reduced it by approximately 26.5%. Under the intervention conditions, the amplitude of EDA fluctuations decreased to approximately 10 ADC units, indicating that the subjects had entered a stable state of deep relaxation, while also validating the effectiveness of the sensors for EDA monitoring. It should be noted that these findings were obtained from a small-scale laboratory validation study and should be interpreted as preliminary evidence of sensor functionality rather than definitive proof of system effectiveness across broader user populations.

### 3.3. EMG Sensor Validation

Another key dimension of physiological parameters is muscle tension, which directly reflects the user’s stress levels and degree of relaxation. As it is not directly controlled by conscious thought, it serves as one of the core indicators for assessing a meditative state. The study employs the Cheez. sEMG sensor to capture this signal. Therefore, conducting reasonable and stable functional validation of the sensor is crucial for ensuring the device’s emotional recognition and adaptive regulation capabilities. The Cheez. sEMG sensor module integrates high-precision signal amplification circuits and noise suppression units. Based on the principle of changes in muscle electrical activity, it non-invasively and in real-time measures electromyographic amplitude (μV). With its compact size, low power consumption, and strong resistance to interference, it is highly suitable for integration into handheld meditation devices for long-term, stable data acquisition. The sensor parameters are as follows (Table 7).

To ensure the validity of the EMG measurements, we strictly controlled the experimental conditions and signal processing parameters.

1. Sensor Placement: The Cheez. sEMG sensor was placed on the flexor carpi radialis muscle (forearm), a site selected for its accessibility and representative reflection of upper-body muscle tension. The skin was cleaned with alcohol wipes prior to attachment to ensure low impedance.
2. Signal Processing: A fourth-order Butterworth low-pass filter with a cutoff frequency of 50 Hz was applied to remove high-frequency noise and motion artifacts. Additionally, a moving average filter (window size = 50 ms) was used to smooth the envelope of the EMG signal. The processed signal was then rectified to calculate the Root Mean Square (RMS) value, which served as the primary metric for muscle activation level.

The raw ADC data was calibrated to reflect the actual electromyographic amplitude (μV). According to the Cheez sEMG sensor specifications (Table 7), the conversion formula is:

(2) $$V=\frac{{ADC}_{raw}}{1024}\times 5.0\times 1,000,000/Gain$$

where the Gain factor is specific to the Cheez sEMG amplifier settings (typically 1000).

**Figure 6:**
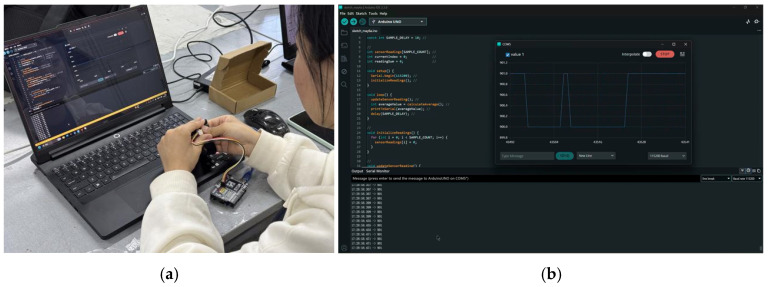
EMG sensor functional validation. (**a**) Sensor functional test; (**b**) test code and data recording.

To effectively validate the sensor’s functionality, the experiment leveraged the principle that auditory pathways directly influence the brain’s limbic system and autonomic nervous system, thereby regulating muscle tension and psychological state. This approach verified EMG signal levels under various physical states, establishing a performance baseline for subsequent applications in meditation scenarios. Sensor functionality validation was conducted in a standard laboratory environment. Subjects sat quietly for 10 min to stabilize their physiological and emotional states. When subjects were in a resting state (with stable physical and emotional conditions and naturally relaxed muscles), the Cheez. sEMG sensor was attached to the inner forearm (an area rich in muscle groups that is easy to secure and free from significant movement interference) (Figure 6).

**Figure 7:**
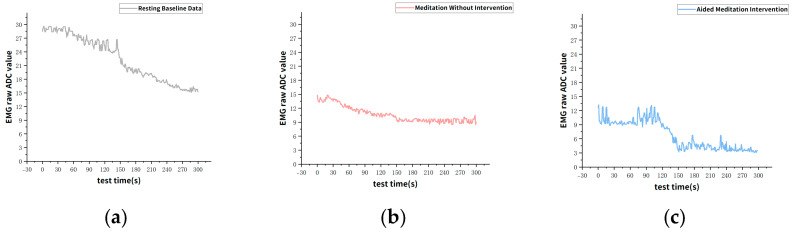
Cheez. sEMG validation data. (**a**) Initial physiological EMG data of subjects wearing the Cheez. sEMG device at rest. (**b**) Physiological EMG data of subjects wearing the Cheez. sEMG device during seated meditation. (**c**) Physiological EMG data of subjects wearing the Cheez. sEMG device during seated meditation with intervention.

Initial physiological baseline data were obtained by continuously collecting resting EMG signal data for 5 min. The experiment ensured good skin contact between the sensor and the skin (adhering to areas rich in muscle tissue and avoiding interference from body hair), connected the data acquisition terminal, and confirmed that the equipment was functioning normally. Based on the resting state baseline data, signal monitoring and intervention experiments were conducted during the meditation state. The subject wore the Cheez. sEMG sensor on the same inner forearm used for the initial physiological baseline measurement (ensuring consistency in the acquisition area, contact pressure, and environmental conditions to minimize systematic error) and begin meditating. During the experiment, participants maintain a quiet, seated meditation posture while real-time EMG sensor data is continuously recorded. During the intervention phase, the assistive device is activated, and EMG data is collected synchronously for 5 min to observe the improvement in muscle relaxation during the meditative state following the intervention (Figure 7), thereby evaluating the baseline monitoring capability of the Cheez. sEMG sensor under normal physiological and environmental conditions.

**Figure 8:**
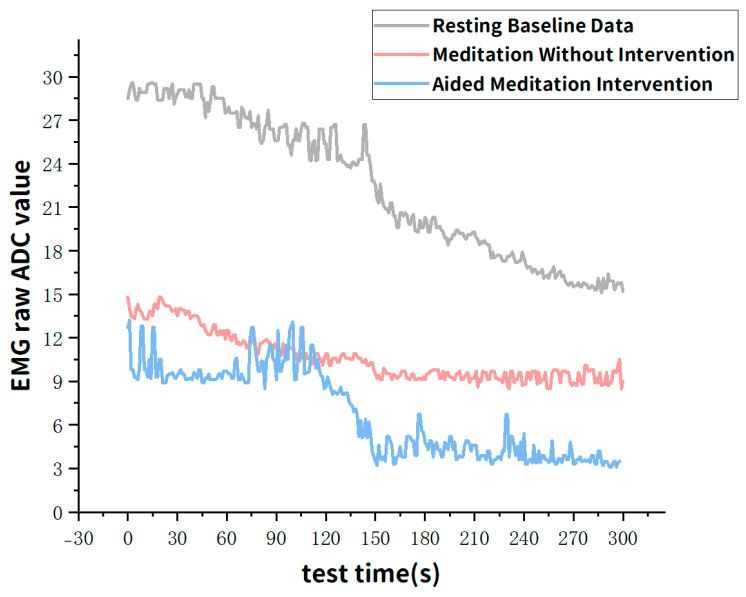
Data after Cheez. sEMG fitting. Fitted EMG data of subjects wearing the Cheez. sEMG device in a resting state under non-intervened and intervened meditation conditions.

During the experiment, the data acquisition terminal continuously recorded physiological data and regulatory behaviors. Subjects were instructed to maintain natural breathing and a relaxed state, avoiding deliberate muscle contraction. After the experiment, the data were organized and fitted based on the aforementioned experimental data (Figure 8).

By comparing data from the three experimental groups (Table 8), we observe a transition from the high mean value of 15.2 μV (corresponding to ~22.5 ADC) in the resting state, decreases to 6.6 μV (~10.7 ADC) during meditation without intervention, and reaches a low mean value of 4.5 μV (~6.8 ADC) in the music-intervention meditation group. Compared to the resting baseline, non-intervention meditation reduced the mean EMG by approximately 56.6%, while intervention meditation further reduced it by approximately 70.3%. Under the intervention conditions, the amplitude of EMG signal fluctuations decreased to approximately 4.3 ADC units, indicating that the subjects had entered a stable state of deep muscle relaxation, while also validating the effectiveness of the sensors for monitoring EMG signals.

The above results indicate that EMG signals can accurately and objectively reflect changes in the degree of muscle relaxation during meditation, serving as a reliable physiological indicator for evaluating meditation efficacy. Traditional meditation can effectively reduce muscle tension and help participants achieve initial physical and mental relaxation, but it is difficult for most participants to quickly enter a state of deep relaxation. These preliminary results suggest that intervention-assisted meditation may contribute to improved relaxation outcomes under the experimental conditions tested. However, further studies involving larger and more diverse participant populations are required to verify the generalizability of these findings.

### 3.4. HR Sensor Validation

The activity state of the autonomic nervous system, particularly instantaneous changes in HR, is also a core indicator for assessing emotional arousal and the depth of meditation. Therefore, conducting reasonable and stable functional validation of the HR sensor is crucial for ensuring the device’s emotional recognition and adaptive regulation capabilities. This study selected the MAX30102 sensor module, which integrates a high-precision PPG unit, signal amplification circuitry, and a noise suppression unit. Based on the principle of photoplethysmography, it measures HR (BPM) non-invasively and in real time. Its compact size, low power consumption, and strong resistance to interference make it highly suitable for integration into handheld meditation devices for long-term, stable data acquisition. The sensor parameters are as follows (Table 9).

To ensure the reliability of the HRV data, a standardized signal processing procedure was applied to the raw PPG signals. First, a 4th-order Butterworth bandpass filter with cutoff frequencies of 0.5–5 Hz was used to remove motion artifacts and baseline wander. Subsequently, the filtered signal was processed using an adaptive threshold-based peak detection algorithm to identify the pulse peaks and calculate the RR intervals. Finally, time-domain HRV metrics, specifically the RMSSD (Root Mean Square of Successive Differences), were computed to quantify the variations in heart rate.

**Figure 9:**
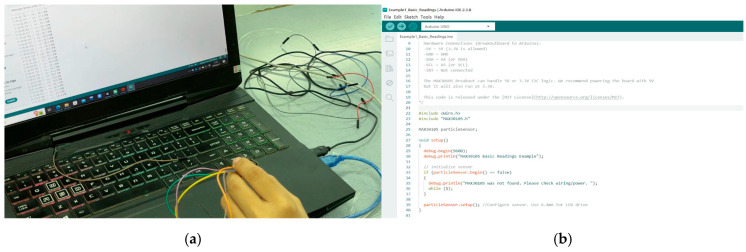
MAX30102 verification experiment. (**a**) Sensor functionality test. (**b**) Test code.

To effectively validate sensor functionality and verify HR variability levels under different conditions, thereby establishing a performance baseline for subsequent meditation applications, the experiment was conducted in a standard laboratory environment to validate sensor functionality. Participants sat quietly for 10 min to stabilize their physiological and emotional states. When participants were in a resting state (with stable physical and emotional conditions), the MAX30102 sensor was attached to their right index finger (Figure 9).

**Figure 10:**
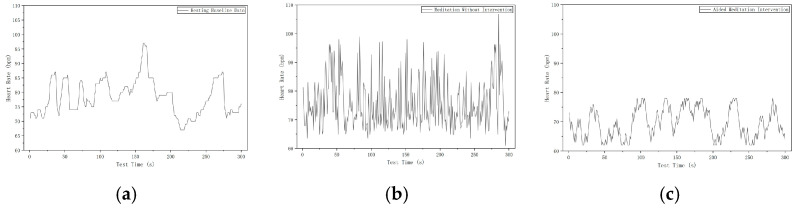
MAX30102 accuracy validation data. (**a**) Initial physiological HR data of subjects wearing the MAX30102 device at rest. (**b**) Physiological HR data of subjects wearing the MAX30102 device during seated meditation. (**c**) Physiological HR data of subjects wearing the MAX30102 device during seated meditation with intervention.

The primary objective of this experimental phase is to obtain HR variability data from participants under different conditions. In the resting state, the experiment continuously collected HR data for 5 min to obtain the subject’s initial physiological baseline data. This assessed the MAX30102 sensor’s basic monitoring capability under normal physiological and environmental conditions, providing a comparable baseline for subsequent performance verification in meditation scenarios. To further validate the sensor’s effectiveness, experiments for traditional meditation monitoring and intervention-regulated monitoring were designed based on the resting state baseline data. Participants wear the MAX30102 sensor on the same finger of the same hand used in the initial physiological baseline measurement experiment (to ensure consistency in the sensing area, contact pressure, and environmental conditions, thereby reducing systematic error) and begin meditating, ensuring good skin contact with the sensor (against the fingertips or wrist). They connect to the data acquisition terminal and confirm that the device is functioning properly. During the experiment, participants sat quietly in meditation while real-time HR sensor data was continuously recorded. During the intervention phase, the assistive device was activated to synchronously collect HR data for 5 min, observing the improvement in relaxation and meditative state following the assistive intervention. Throughout the experiment, the data acquisition terminal continuously recorded physiological data and behavioral adjustments, with participants maintaining natural breathing and a relaxed state (Figure 10).

**Figure 11:**
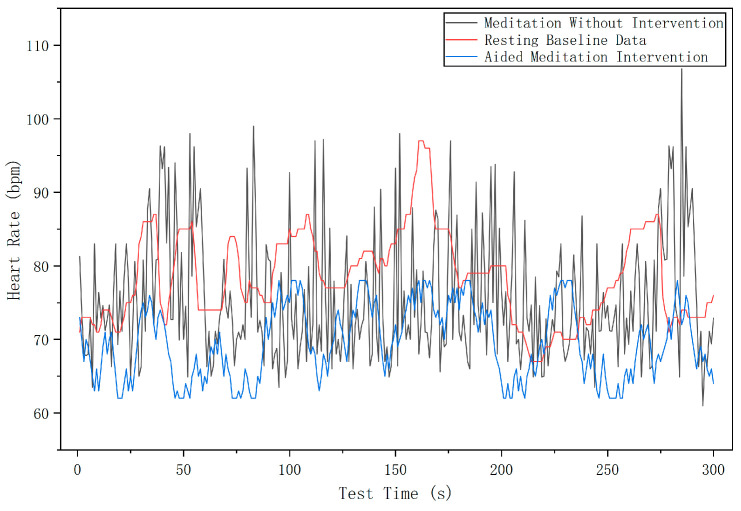
Data after MAX30102 fitting. Fitted HR data of subjects wearing the MAX30102 in a resting state under non-intervened and intervened meditation conditions.

By comparing the experimental data from the three groups (Table 10). From the high mean HR (~75 BPM) and significant variability (~8 BPM) in the resting state, to the moderate mean HR (~69 BPM) and substantial variability (~22 BPM) in non-intervention meditation, and finally to the low mean HR (~65 BPM) and minimal variability (~4 BPM) in music-intervention meditation, the above comparison reveals a non-monotonic yet meaningful pattern of physiological changes. Specifically, at rest, the HR stabilized at 70–78 BPM, reflecting a state of wakeful arousal; during unguided meditation, the mean HR dropped to 69 BPM, a decrease of approximately 8.0% from baseline, but the range of HR variability expanded to 22 BPM, with the range widening to 45–87 BPM, suggesting that the body entered a state of initial relaxation accompanied by certain physiological regulatory fluctuations; In contrast, during music-assisted meditation, the mean HR further decreased to 65 BPM (a relative decrease of approximately 13.3% from baseline), with the range of variation narrowing to just 4 BPM and the range compressing to 63–67 BPM, indicating a state of deep relaxation and high physiological stability (Figure 11).

The above results indicate that HR signals can sensitively reflect changes in the degree of relaxation during meditation and serve as a reliable indicator for evaluating meditation effectiveness. Unguided meditation can effectively reduce sympathetic nervous system arousal and facilitate initial relaxation; however, increased HR variability suggests that physiological regulation has not yet stabilized. In contrast, the soothing music intervention further reduced HR and significantly improved physiological stability (variability decreased from 22 BPM to 4 BPM), helping participants rapidly enter and maintain a deep relaxation state. The MAX30102 HR monitoring device used in this experiment accurately captures subtle changes in physiological state and demonstrates excellent performance and reliability.

### 3.5. Verification of System Closed-Loop Response

To verify the end-to-end real-time performance and accuracy of the haptic meditation enhancement device—from physiological signal acquisition and state classification to multimodal feedback output—a closed-loop response validation experiment was designed. This validation focused on evaluating the system’s response latency to simulated emotional fluctuations, the consistency between feedback triggers and the user’s subjective experience, and the coordinated reliability of multi-level feedback (light, sound, and scent).

**Figure 12:**
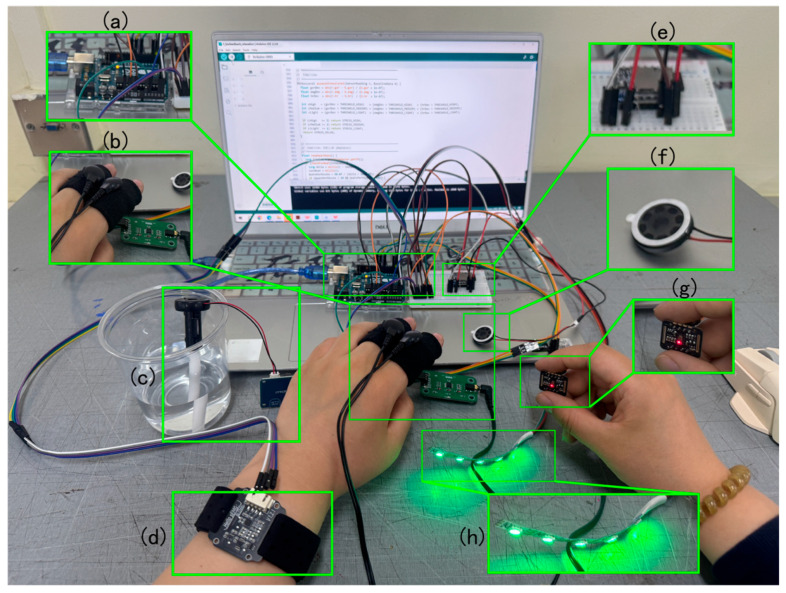
Prototype of the haptic meditation enhancement device. (**a**) Arduino UNO control board. (**b**) SICHIRAY GSR V galvanic skin response sensor (**c**) HE-30 nebulizer module. (**d**) Cheez. sEMG electromyography sensor. (**e**) DF Player Mini audio playback module. (**f**) Audio speaker. (**g**) MAX30102 HR sensor. (**h**) WS2812B programmable RGB LED.

The complete prototype consists of two components: a physiological monitoring unit worn by the subject (including EEG sensors, EMG sensors, and HR sensors) and the main body of the handheld output device (integrating an audio playback module, programmable RGB LEDs, and a misting module). These two components work in concert to form a closed-loop system (Figure 12). During the experiment, subjects performed simulated meditation tests in a quiet environment. Based on physiological signals collected from the three sensor channels, the system fused the data to determine emotional arousal and relaxation levels, automatically triggering different levels of feedback responses (light, music, and misted scents) on the handheld device, while simultaneously recording data at each stage.

**Figure 13:**
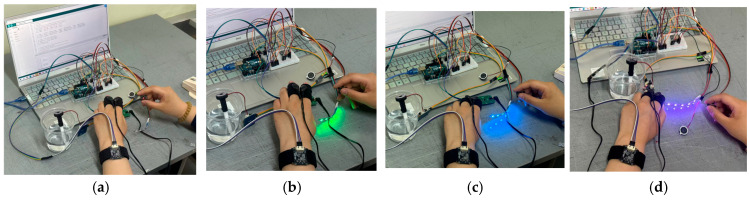
Operational status of the haptic meditation enhancement device prototype system. (**a**) Initial startup state, sensor self-test (**b**) System monitoring of the subject’s normal emotional state (**c**) System monitoring of slight fluctuations in the subject’s psychological state (**d**) System monitoring of significant fluctuations in the subject’s psychological state.

First, the initial operating state of the device is recorded. At this point, the device has just been powered on and is in the initialization and calibration phase; no valid physiological signals have been detected yet, and the system is in standby mode. In this state, the device indicator light is off, and neither the integrated audio module nor the misting module is active. Once the sensors complete their self-tests and begin to output stable data, the system automatically enters monitoring mode (Figure 13a).

When the subject is in a normal emotional state, the parameters collected by the three sensors are all within the normal range. Specifically, at rest, the mean ADC value for skin conductance is approximately 22.5, the mean ADC value for electromyography is approximately 22.5, and the HR is stable within the 70–78 BPM range. The system’s fusion algorithm determines that the mood is stable and no intervention is required. The device indicator light turns green to indicate a normal status, and both the integrated audio and misting functions remain off. In this state, the system continues to monitor but does not actively intervene, reflecting the device’s principle of low-interference operation when not necessary (Figure 13b).

When the subject experiences slight mood fluctuations (such as environmental noise or wandering thoughts), recognizable deviations begin to appear in the sensor data. The EDA signal rises slightly by approximately 5–10%, the HR variability increases from ~8 BPM at rest to ~12 BPM, while changes in the EMG signal are not yet significant. The system’s fusion algorithm classifies this as a mild arousal/pre-stress state and automatically triggers a Level 1 intervention response: the device indicator light switches from green to blue, and the integrated audio module begins playing soothing natural white noise or light music (Figure 13c). During this phase, the misting module remains off. Data shows that within approximately 30 s of music playback, the subject’s HR variability gradually narrows, indicating that the Level 1 intervention has begun to take effect.

During states of intense emotional fluctuation, when the subject actively simulated pronounced anxiety or mental agitation (such as recalling stressful events or deliberately accelerating breathing), all three sensor parameters exhibited significant shifts. The mean skin conductance rose from ~22.5 ADC at rest to ~35 ADC, a relative increase of approximately 55%; the mean EMG value rose from ~10.7 ADC to ~18 ADC, indicating a marked increase in physical tension; the mean HR jumped from ~69 BPM to ~87 BPM, with instantaneous peaks exceeding 95 BPM, and the amplitude of HR fluctuations expanded to approximately 22 BPM. In response to this state of high arousal and high tension, the system automatically triggered a secondary intervention following fusion analysis: the device indicator light turned purple; the integrated audio module continued playing music while appropriately slowing the tempo to enhance the calming effect; and the integrated misting module activated simultaneously, releasing a liquid mist (such as an essential oil mist containing lavender or chamomile) to help alleviate emotional stress (Figure 13d). Experimental records show that within 60 s of the dual intervention being initiated, the subjects’ mean HRs gradually returned to ~75 BPM, and both skin conductance and electromyography signals began to show a downward trend, indicating that the integrated device’s multimodal environmental regulation capabilities effectively helped the subjects transition from a state of high tension to a state of relaxation.

Throughout the entire experiment, the integrated device’s responses fully aligned with the preset logic, with no instances of false triggers, response delays, or malfunctioning components. The real-time outputs from the three sensor channels—skin conductance, electromyography, and HR—maintained a stable correspondence with their respective trigger thresholds, and the fusion classification algorithm accurately distinguished between three distinct physiological patterns: normal state, mild fluctuations, and severe fluctuations. The response delay for the device’s indicator light color changes was less than 0.5 s; music playback and switching via the integrated audio module were smooth and uninterrupted; the atomization trigger of the integrated misting module exhibited good synchronization; and all actuators operated seamlessly in unison within the unified housing.

In summary, the integrated haptic meditation enhancement device proposed in this study demonstrated accuracy and reliability during systematic closed-loop response validation. This integrated device can automatically adapt to a gradient feedback strategy—ranging from “green (no intervention)” to “blue (Level 1 intervention: music only)” to “purple (Level 2 intervention: music + misting)”—based on subtle changes in the user’s physiological state, successfully achieving a complete closed-loop control system spanning physiological sensing, state classification, and environmental adjustment. The experimental results fully demonstrate that this integrated device possesses excellent real-time performance, sensitivity, and system integration, making it suitable for practical applications in handheld meditation-support and emotional intervention.

### 3.6. Illustration of the Handheld Meditation Device’s Effects

To visually demonstrate the expected operational state and intervention effects of the haptic meditation enhancement device in actual use, this section presents the device’s performance through schematic diagrams of typical usage scenarios.

**Figure 14:**
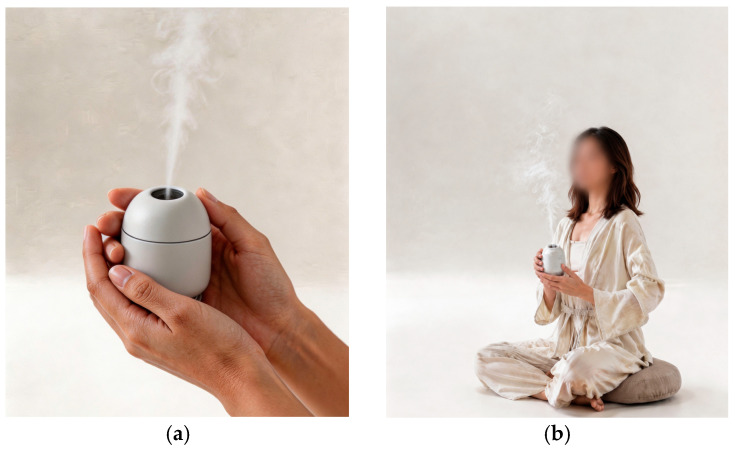
Schematic illustration of the haptic meditation enhancement device. (**a**) Handheld state. (**b**) Usage scenario.

The device features a handheld, integrated design that combines a GSR sensor (GSR V2), an EMG sensor (Cheez sEMG), and a HR sensor (MAX30102). The user holds the device naturally, with their fingers in contact with the sensor electrodes. The bottom of the device is equipped with a programmable RGB LED indicator and an audio output port, while the top features an odor diffusion outlet. The device weighs approximately 120 g, making it comfortable to hold for extended periods, as shown in Figure 14.

The threshold ranges adopted in the hierarchical decision model were selected as engineering design parameters based on preliminary pilot observations, practical system-testing experience, and physiological fluctuation patterns reported in previous biofeedback and stress-monitoring studies [49,50]. Since substantial inter-individual variability exists in physiological responses, the proposed system evaluates changes relative to each user’s personalized baseline rather than relying on fixed absolute physiological values. The current threshold settings are intended to support prototype-level adaptive intervention and have not yet been optimized through large-scale statistical validation. Future studies involving larger participant populations will be conducted to further refine and validate these decision boundaries.

When the user is in a calm state (all three physiological indicators are within ±10% of the individual baseline), the device maintains only the basic breathing light (slow green flash) and does not apply additional interventions. When mild tension or distraction is detected (a single physiological indicator exceeds the baseline by 10–25%), the light switches to blue and enters a breathing gradient mode to guide the user in regulating their breathing rhythm. When any two physiological indicators simultaneously exceed the baseline by 25–40%, the state is classified as moderate tension. In this case, the device plays soothing piano music (at approximately 40 dB) in addition to the lighting, helping the user shift their attention and reduce arousal levels. When all three indicators exceed the baseline by more than 40%, the state is classified as high stress or anxiety, and the device simultaneously triggers a combination of light (soft purple light), sound (natural white noise), and scent (lavender or citrus fragrance) to promote rapid relaxation through the synergistic action of multiple sensory channels.

## 4. Field Measurement Results

### 4.1. Analysis of Physiological Data

Psychology and psychological data were analyzed using paired-samples *t*-tests to compare pre- and post-intervention measurements within each group. The psychological assessment results (SAS and PSS) were summarized as mean (M) ± standard deviation (SD). Effect sizes were calculated using Cohen’s d. Statistical significance was defined as *p* < 0.05. All analyses were performed using SPSS software (IBM SPSS Statistics 26.0, IBM Corporation, Armonk, NY, USA).

**Figure 15:**
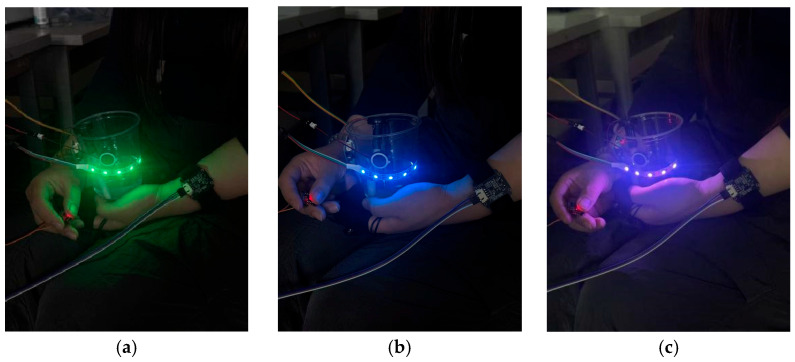
Subject status feedback. (**a**) Normal state. (**b**) State of mild psychological fluctuation (**c**) State of intense psychological fluctuation.

To verify the device’s effectiveness in regulating human physiological states, the experiment compared changes in physiological parameters before and after meditation between the experimental group (using the haptic meditation enhancement device) and the control group (performing traditional closed-eye meditation without device assistance). Key indicators such as electrodermal activity (EDA), HR (HR), and blood oxygen saturation (SpO_2_) were systematically collected. Figure 15 illustrates the participants’ usage feedback.

The experimental group’s average EDA value before using the device was 22.5 ADC. During a “state of intense fluctuation” automatically identified by the system, EDA rose sharply to 35 ADC; however, within 60 s after the device automatically triggered the intervention (light + music + lavender essential oil spray), EDA dropped significantly to ~18 ADC. Compared to the control group, under the same emotional induction, participants not wearing the device took approximately 2.5 times longer for their skin conductance to return to baseline, and the reduction in skin conductance reached only 60% of that observed in the experimental group. This indicates that the device possesses the ability to rapidly modulate sympathetic nervous system activity.

The resting HR in the experimental group stabilized at 70–78 BPM. When mild signs of stress appeared, the range of HR fluctuations expanded from ±8 BPM to ±12 BPM; under high arousal conditions, the mean HR surged to 87 BPM, with peaks reaching over 95 BPM. Following device intervention, the HR rapidly returned to ~75 BPM within approximately 60 s, with the fluctuation range narrowing to ±10 BPM. In the control group, without regulatory feedback, the elevated HR state persisted for an average of over 180 s and struggled to return to baseline levels on its own.

Under conditions of intense stress (such as simulated anxiety or recalling stressful events), participants’ average electromyographic (EMG) values rose from 10.7 ADC at rest to 18 ADC due to disrupted breathing rhythms, indicating a significant increase in physical tension. After the device initiated the second-stage intervention, EMG values decreased significantly within 60 s, gradually approaching resting levels. In contrast, EMG signals in the control group declined slowly, and some participants even maintained high muscle tension after the meditation session ended.

Furthermore, analysis of Heart Rate Variability (HRV) corroborated these findings. The RMSSD values increased significantly during the intervention (from M = 32.45 ms, SD = 12.30 to M = 48.62 ms, SD = 15.10), indicating enhanced parasympathetic activity (t(29) = 5.82, *p* < 0.001, Cohen’s d = 1.15, 95% CI [10.52, 21.82]). Similarly, the LF/HF ratio decreased significantly (from M = 2.85, SD = 1.10 to M = 1.65, SD = 0.85; t (29) = −6.15, *p* < 0.001, Cohen’s d = −1.25, 95% CI [−1.58, −0.82]), suggesting a shift towards physiological relaxation consistent with the reduced sympathetic arousal observed in GSR and HR.

Overall, the physiological indicators in the experimental group exhibited a faster and smoother return to baseline following the device intervention, validating the device’s significant adaptive regulatory effect on human physiological states.

### 4.2. Psychological State Assessment

Standardized psychological scales (including the SAS and the PSS) were used to conduct pre- and post-meditation comparisons between the experimental and control groups.

As shown in Table 11, both groups demonstrated reductions in anxiety and perceived stress after meditation. The experimental group exhibited larger reductions in both SAS and PSS scores than the control group. Effect sizes (Cohen’s d) are reported to facilitate interpretation of the magnitude of the observed changes.

The experimental group’s average SAS score before meditation was 48.53, indicating mild anxiety. After completing 15 min of device-assisted meditation, the average SAS score decreased to 29.93, representing a reduction of 38.3%. The control group, which performed conventional meditation for the same duration, showed a decrease in SAS scores from 47.87 to 39.53, corresponding to a reduction of 17.4%. These findings suggest that the experimental group experienced greater improvements in anxiety symptoms following the intervention. Similarly, the experimental group’s average PSS score decreased from 28.60 before meditation to 16.33 after meditation, representing a reduction of 42.9%. In the control group, the average PSS score decreased from 27.47 to 22.40, corresponding to a reduction of 18.2%. The larger reductions observed in the experimental group indicate that the device-assisted meditation approach may provide greater benefits for stress reduction than conventional meditation. These findings are consistent with the physiological outcomes and suggest that real-time physiological feedback and multimodal intervention may facilitate deeper psychological engagement and contribute to reductions in anxiety and perceived stress.

## 5. Conclusions

The physiological results demonstrate that our system can effectively induce a relaxation response in users. Analysis of the experimental data revealed significant reductions in key physiological indicators—EDA, EMG, and HR—within approximately 60 s of intervention, with effect sizes (Cohen’s d) exceeding 1.0, indicating a strong impact on the autonomic nervous system. The consistency between these physiological improvements and the subjective psychological measures (SAS and PSS scores) further validates the effectiveness of the HMED system. Notably, the multimodal intervention approach (light, sound, and scent) appears to offer advantages over single-modality techniques, suggesting that the synergistic effect is crucial for rapidly guiding beginners into a deep meditative state.

Compared to existing standalone meditation apps or wearable physiological monitoring bands, this device offers two key differentiating advantages. First, it employs passive, non-interactive intervention. Without requiring users to actively tap the screen or switch modes, the device spontaneously “reads” emotions based on physiological signals and executes interventions, significantly reducing distractions to the user’s focus. Second, it utilizes parallel multi-sensory channels. The device integrates visual (lighting), auditory (music), and olfactory (essential oil diffusion) stimuli into a single experience, overcoming the limitations of traditional audio-only meditation. This immersive environment actively shapes the user’s state—a capability that cannot be achieved through data logging or voice guidance alone.

This study has the following limitations. First, the actuation latency of the olfactory module (2–3 s) is inherent to the current atomization hardware and is significantly longer than the electronic signal processing loop. In addition, the current validation study involved a relatively small group of healthy participants within a limited age range, which may restrict the generalizability of the findings to broader user populations. Therefore, the current results should be interpreted primarily as evidence of prototype feasibility and functional effectiveness rather than proof of broad applicability. Furthermore, larger and more diverse participant groups will be recruited, and longer-term evaluations under real-world conditions will be conducted to further assess the effectiveness, personalization capability, and generalizability of the proposed system. Second, the experimental setting was a controlled, soundproof laboratory, which differs from the complex environmental noise interference found in actual home or office settings. Third, and most importantly, our current validation primarily focuses on the immediate pre/post physiological changes during brief sessions. While the device demonstrates strong short-term efficacy, we acknowledge that this initial study has not yet established the long-term sustained benefits, the process of habit formation, or the long-term reduction in chronic stress. We agree with the reviewer that these are crucial metrics for evaluating a meditation-support device. Furthermore, although comprehensive long-term field tests were beyond the scope of this initial study, practical usability was carefully considered during the design phase. To ensure comfort and wearability, the device was designed with a compact and lightweight handheld form factor, utilizing skin-friendly materials to minimize fatigue during repeated use. Regarding battery and runtime behavior, the system architecture was optimized for low power consumption to support extended meditation sessions without frequent recharging. Finally, the cost-effective and modular hardware design was intentionally chosen to enhance user acceptance and facilitate future robustness testing in realistic, uncontrolled environments.

Additionally, while this study demonstrates the efficacy of the multimodal system, the specific contribution of each individual modality (music vs. light vs. scent) was not isolated in this iteration. Future work will employ factorial designs to quantify the unique impact of each sensory channel.

Based on these limitations and user feedback, future work will focus on hardware optimization, algorithm evolution, scenario expansion, and human–machine empathy. Specifically, we plan to conduct longitudinal studies to monitor user engagement over weeks or months, in order to validate the device’s ability to promote habit formation and sustained stress reduction. By developing a desktop-level hands-free docking station and a wearable wrist-mounted version, we aim to enhance comfort during prolonged meditation sessions. By incorporating deep learning models, we will enhance the system’s ability to recognize specific emotional states (such as sudden panic or extreme fatigue) and reduce false trigger rates caused by individual differences. Furthermore, the system can be adapted to other immersive scenarios—such as driver fatigue intervention, workplace stress management, and insomnia treatment—to further test its stability.

In summary, this integrated haptic meditation enhancement device has successfully demonstrated that multi-modal sensor fusion and adaptive environmental interventions can effectively regulate human physiological and psychological states, offering promising application prospects and value for further development.

## Figures and Tables

**Table 1:** Comparison of Mainstream Embedded Development Platform Categories.

Platform Category	Key Advantages	Major Shortcomings	Suitability for This Project
51 Series	Extremely low cost, abundant resources	Weak performance, few analog interfaces, no native PWM multi-channel output	Not applicable
STM32 Series	Powerful performance, rich peripherals, high reliability	Complex development environment, steep learning curve, long prototyping cycle	Redundant performance, high development costs
ESP Series	Native Wi-Fi/Bluetooth, high IoT integration	Limited ADC precision; weaker ecosystem for driving analog sensors compared to Arduino	Strong wireless capabilities, but limited applicability for analog data acquisition scenarios
Arduino Series	Low development barrier, comprehensive sensor library ecosystem, ample analog interfaces, and extensive community support	Clock frequency and memory resources are lower than those of the STM32	Highly suitable, with significant overall advantages

**Table 2:** Arduino UNO R3 Technical Specifications.

Specifications	Value
Microcontroller	ATmega328P
Operating Voltage	5 V
Input Voltage (Recommended)	7–12 V
Digital I/O Pins	14 (6 of which support PWM output)
Analog Input Channels	6 (10-bit precision ADC)
Continuous current per I/O pin	20 mA
Flash memory	32 KB (of which 0.5 KB is used for the bootloader)
SRAM	2 KB
EEPROM	1 KB
Clock frequency	16 MHz
Dimensions	68.6 mm × 53.4 mm
Weight	Approx. 25 g

**Table 3:** Response Times of Sensors and Actuator Modules.

Module Category	Function Type	Model	Response Time
Main Control Board	Microcontroller	Arduino UNO R3	1–8 ms
Input Sensor	GSR Sensor	SICHIRAY GSR V2 (Wuxi Sichiray Technology Co., Ltd, Wuxi, China)	50–100 ms
Input sensor	HR Sensor	MAX30102	5–20 ms
Input sensor	EMG sensor	Cheez. sEMG (Cheez Lab, Shanghai, China)	2–5 ms
Output module	Programmable RGB LED	WS2812B (WorldSemi, Shenzhen, China)	1–3 ms
Output Module	Audio Playback Module	DF Player Mini (DFRobot, Shanghai, China)	20–50 ms
Output Module	Odor Diffusion Module (Mist-Driven)	HE-30 (Shenzhen Scentsea Technology Co., Ltd., Shenzhen, China)	2–3 s

Note: The response time of the odor diffusion module (2–3 s) is an order of magnitude slower than electronic sensors. This study explicitly separates the high-speed physiological detection loop (<200 ms) from the hardware-specific actuation delays to ensure scientific accuracy.

**Table 4:** Device Components.

Component Category	Function Type	Model	Price
Main Control Board	Microcontroller	Arduino UNO R3	$15.00
Input Sensors	GSR Sensor	SICHIRAY GSR V2	$6.80
Input Sensor	HR Sensor	MAX30102	$2.30
Input Sensor	EMG Sensor	Cheez. sEMG	$12.00
Output Module	Programmable RGB LED	WS2812B	$3.00/m
Output Module	Audio Playback Module	DFPlayer Mini	$5.00
Output Module	Scent Diffusion Module (Mist Driver)	HE-30	$0.73

**Table 5:** Skin Conductance Sensor Parameters.

	** 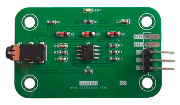 **
Sensor Model	SICHIRAY GSR V2
Operating Voltage	3.3–5 V
Operating Temperature	−10–60 °C
Applicable Scenarios	Resting state, meditation, emotional fluctuations, daily relaxation monitoring
Dimensions	48 × 30 mm
Price	$4.2
RR measurement range	0–5 μS
Accuracy	±0.1 μS

**Table 6:** Statistics of Key Indicators.

Evaluation Indicators	Resting Baseline Data	Meditation Without Intervention Data	Meditation with Intervention Data
Mean GSR Value (μS)	2.43	~1.93	~1.75
Fluctuation Amplitude (μS)	1.00	0.80	~0.10
Relative Decrease from Baseline	—	~20.6%	~26.5%
Physiological Interpretation	Awake/Active State	Initial Relaxation State	Deep Relaxation State

**Table 7:** EMG Sensor Parameters.

	** 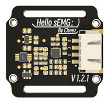 **
Sensor Model	Cheez. sEMG
Operating Temperature	−10–60 °C
Applicable Scenarios	Resting state, meditation, muscle relaxation, muscle tension monitoring
Dimensions	42 × 45 mm
Price	USD 4.2
EMG measurement range	0–100 μV
Accuracy	±1 μV
Operating Temperature	−10–60 °C

**Table 8:** Statistics on Key Indicators.

Evaluation Indicators	Resting Baseline Data	Meditation Without Intervention Data	Meditation with Intervention Data
Mean Signal Value (μS)	15.2	~6.6	~4.5
Fluctuation Amplitude (μS)	9.8	~4.3	~2.9
Relative Decrease from Baseline	—	~56.6%	~70.3%
Physiological Interpretation	Tension/Activated State	Relaxation State	Deep Relaxation State

**Table 9:** Heart Rate Sensor Parameters.

	** 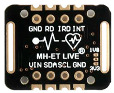 **
Sensor Model	MAX30102
Operating Voltage	2.7–3.3 V
Operating Temperature	−40–80 °C
Applicable Scenarios	Resting state, daily activities, hypoxic environments, and other multiple states
Dimensions	15.6 × 8.3 × 1.55 mm
Price	$3
HR measurement range	30–240 BPM
Accuracy	±2 BPM

**Table 10:** Statistical Analysis of Key Indicators.

Evaluation Indicators	Resting Baseline Data	Meditation Without Intervention Data	Meditation with Intervention Data
Mean HR (BPM)	~75	~69	~65
HR Variability (bpm)	~8	~22	~4
Value Range	70–78	45–87	63–67
Relative Decrease from Baseline	—	~8.0%	~13.3%
Physiological Interpretation	Awake/Active State	Initial Relaxation State	Deep Relaxation State

**Table 11:** Psychological assessment results before and after the intervention.

**Mea** **s** **ure**	**Group**	**Pre-Intervention (M ± SD)**	**Post** **-** **Intervention (M ± SD)**	**Mean Change**	**t**	***p***	**Cohen’s d**
SAS	Experimental (*n* = 15)	48.53 ± 5.50	29.93 ± 3.13	−18.60	12.18	<0.001	3.15
SAS	Control (*n* = 15)	47.87 ± 5.63	39.53 ± 4.36	−8.33	3.56	0.003	0.92
PSS	Experimental (*n* = 15)	28.60 ± 4.05	16.33 ± 2.87	−12.27	10.76	<0.001	2.78
PSS	Control (*n* = 15)	27.47 ± 4.03	22.40 ± 3.46	−5.07	2.89	0.012	0.75

## Data Availability

The data presented in this study are available on request from the corresponding author. The data are unavailable due to privacy restrictions.
